# A Case of Wunderlich Syndrome Caused by Pyelonephritis Due to Klebsiella pneumoniae

**DOI:** 10.7759/cureus.70131

**Published:** 2024-09-24

**Authors:** Hideya Itagaki, Takuro Hagino, Katuhiko Suzuki

**Affiliations:** 1 Department of Emergency and Disaster Medicine, Tohoku Medical and Pharmaceutical University Hospital, Miyagi, JPN; 2 Department of Neurology, Tohoku Medical and Pharmaceutical University Hospital, Sendai, JPN; 3 Department of General Surgery, Honjoudaiichi Hospital, Yurihonjou, JPN

**Keywords:** klebsiella pneumoniae, pyelonephritis, retroperitoneal abscess, spontaneous subcapsular renal hematoma, wunderlich syndrome

## Abstract

Wunderlich syndrome, characterized by spontaneous nontraumatic renal hemorrhage, is a rare but severe condition often presenting with Lenk's triad: acute abdominal pain, flank mass, and hypovolemic shock. While typically caused by neoplastic or vascular conditions, infection-induced Wunderlich syndrome is uncommon. This case report details an 80-year-old woman who developed Wunderlich syndrome secondary to pyelonephritis caused by *Klebsiella pneumoniae*. The patient presented with septic shock and was diagnosed with left subcapsular renal haematoma. Despite initial antimicrobial therapy, the patient's condition deteriorated, requiring surgical drainage. This case emphasizes the importance of considering surgical intervention in addition to antimicrobial treatment in managing Wunderlich syndrome, especially in patients with pre-existing conditions like diabetes mellitus, which increases the risk of severe complications.

## Introduction

Wunderlich syndrome is characterized by nontraumatic spontaneous renal hemorrhage that occurs in the subcapsular or perirenal space and typically presents with Lenk's triad: acute lateral abdominal pain, flank mass, and hypovolemic shock [1,2]. Causes of Wunderlich syndrome include neoplastic, coagulopathy, ruptured abdominal aortic aneurysm, renal vascular disease, dysuria, nephritis, and antithrombotic medications, but infection is rare [1,3,4]. In this case, we experienced a case of Wunderlich syndrome caused by pyelonephritis due to *Klebsiella pneumoniae*.

## Case presentation

**Figure 1:**
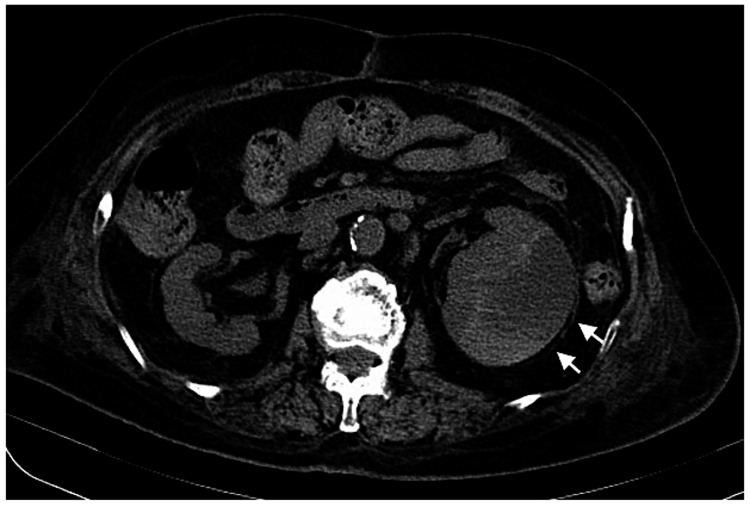
CT scan showed an enlarged left renal subcapsular hematoma (white arrows).

An 80-year-old woman is evaluated in the emergency department for fever with chills. She had been suffering from anorexia for a week. On the day of the visit, she developed a fever with chills and vomiting and was sent to our hospital for emergency care. When she came to our hospital, her vital signs were blood pressure of 83/42 mmHg, pulse of 123 bpm, and temperature of 39.2°C. She had fever and shock. Her medical history included hypertension, hyperlipidemia, diabetes mellitus, myocardial infarction, and rheumatoid arthritis, and she was treated with carvedilol, isosorbide nitrate, aspirin, pitavastatin calcium hydrate, febuxostat, esomeprazole magnesium hydrate, polaprezinc, magnesium oxide, and prednisolone, urapidil, linagliptin, and subcutaneous insulin for diabetes. Suspecting septic shock, blood tests, blood/urine cultures, and imaging studies were performed. On physical examination, vital signs showed shock vital（blood pressure 83/42 mmHg, pulse rate 123/min）and hypoxemia（oxygen saturation 93% on ambient air). Laboratory tests showed an elevated inflammatory response (white blood cell count 9500/µL, C-reactive protein level 13.57 mg/dL) and decreased renal function (blood urea nitrogen 24.4 mg/dL and creatinine 1.19 mg/dL). Computed Tomography [CT] scan showed an enlarged left renal subcapsular hematoma (Figure 1).

**Figure 2:**
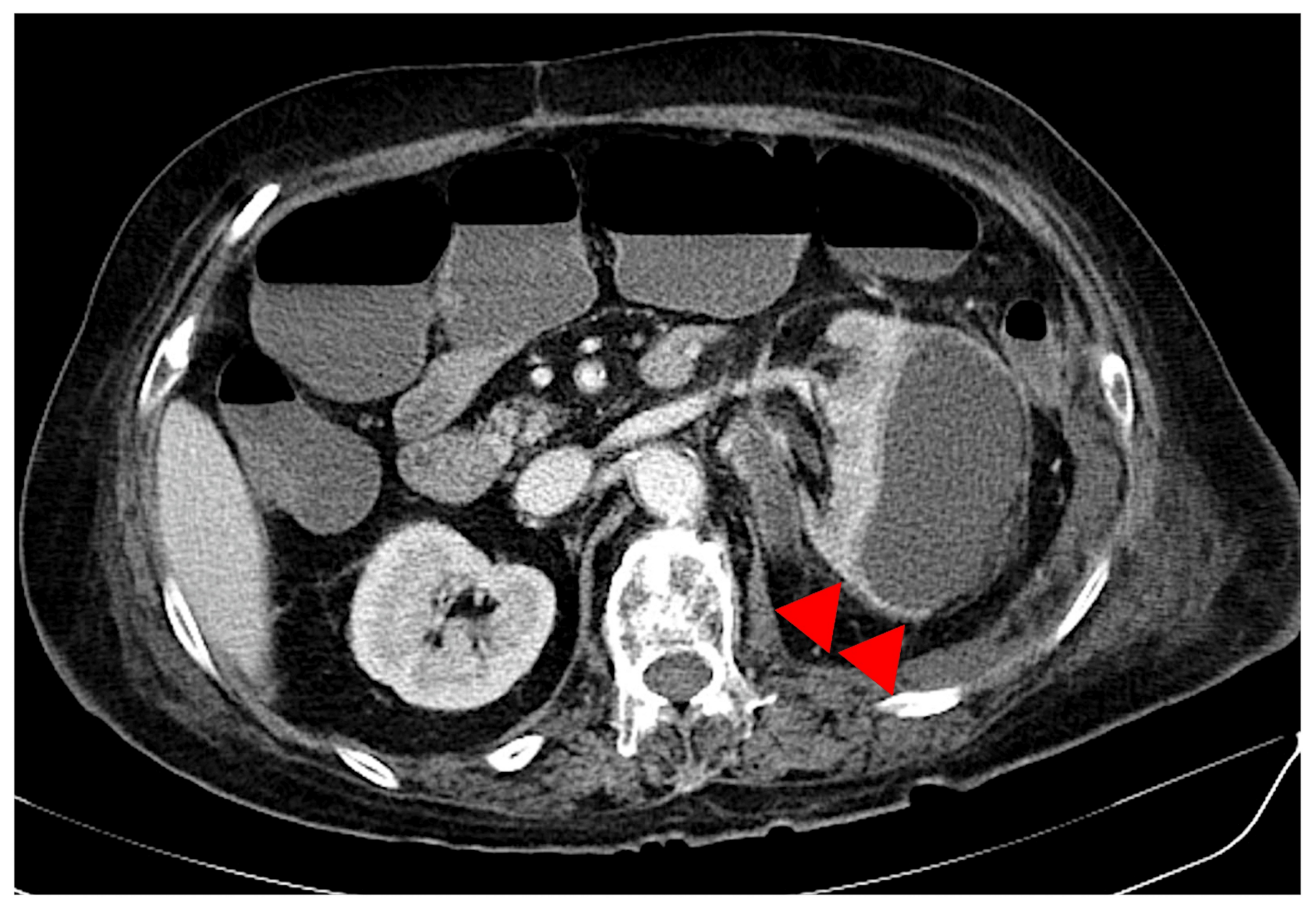
CT scan revealed a contrast effect suspicious for left subrenal capsular hematoma infection (red arrowheads).

Based on the above, the patient was in septic shock with left subrenal capsular hemorrhage and was admitted to the hospital at the same time. Antimicrobial tazobactam/piperacillin (4.5 g x 2 times/day intravenously) and a hypertensive drug (dopamine) were administered from the day of admission. On the third day of admission, *Klebsiella pneumoniae *was detected in blood/urine culture, and the patient was diagnosed with acute pyelonephritis due to *Klebsiella *bacteremia and left subrenal capsular hemorrhage from pyelonephritis: Wunderlich syndrome. So, we suspected septic shock and implemented hospitalization. Subsequently, despite continued antimicrobial therapy, the patient continued to have a prolonged inflammatory response. On day 59 of hospitalization, the patient developed left-sided abdominal pain with tenderness, and a contrast-enhanced CT scan was performed. The contrast-enhanced CT scan revealed a contrast effect suspicious for left subrenal capsular hematoma infection (Figure 2).

**Figure 3:**
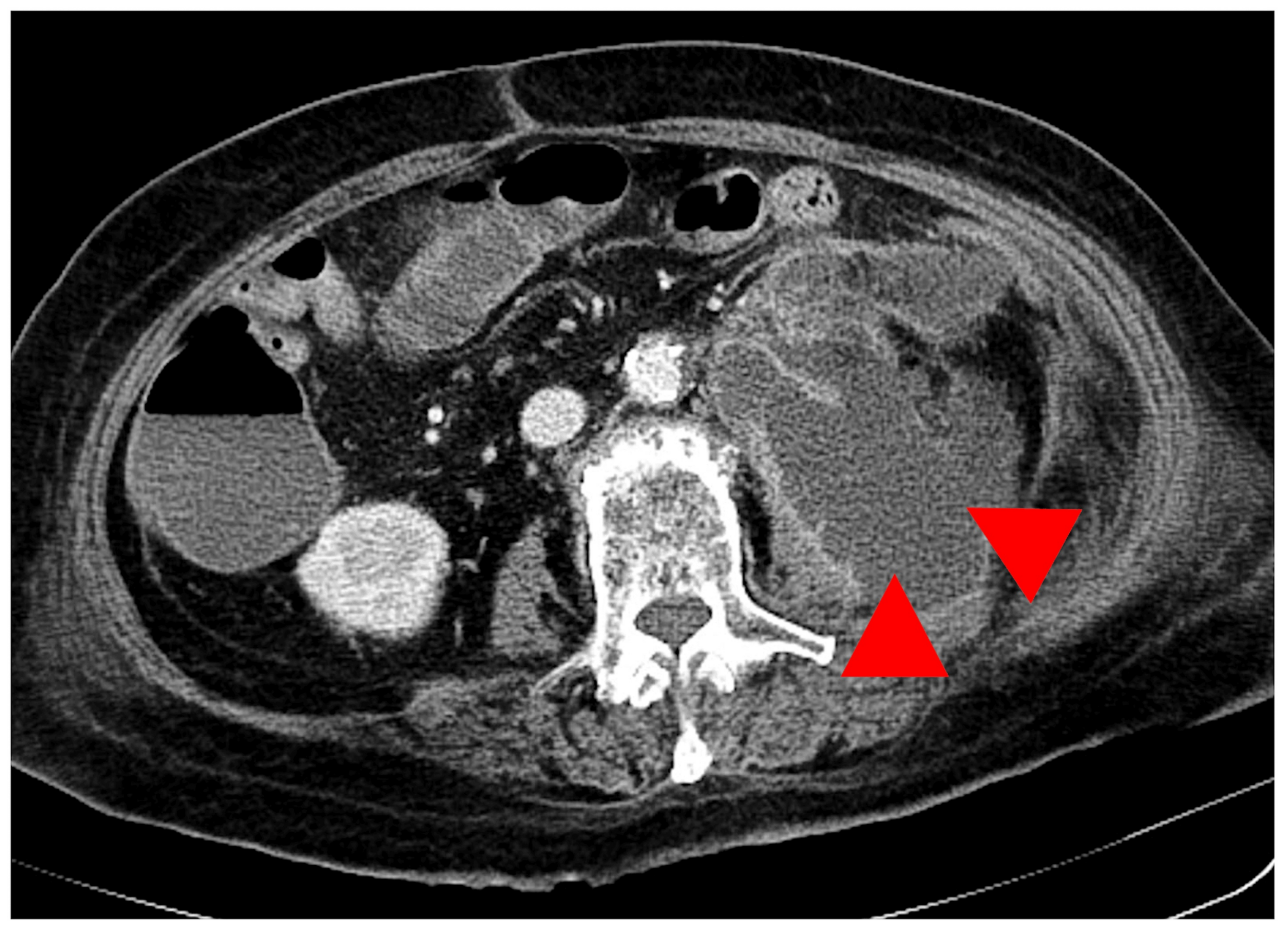
CT scan revealed a retroperitoneal abscess (red arrowheads).

The contrast-enhanced CT scan also revealed a retroperitoneal abscess (Figure 3) in contact with the erector spinae muscle.

**Figure 4:**
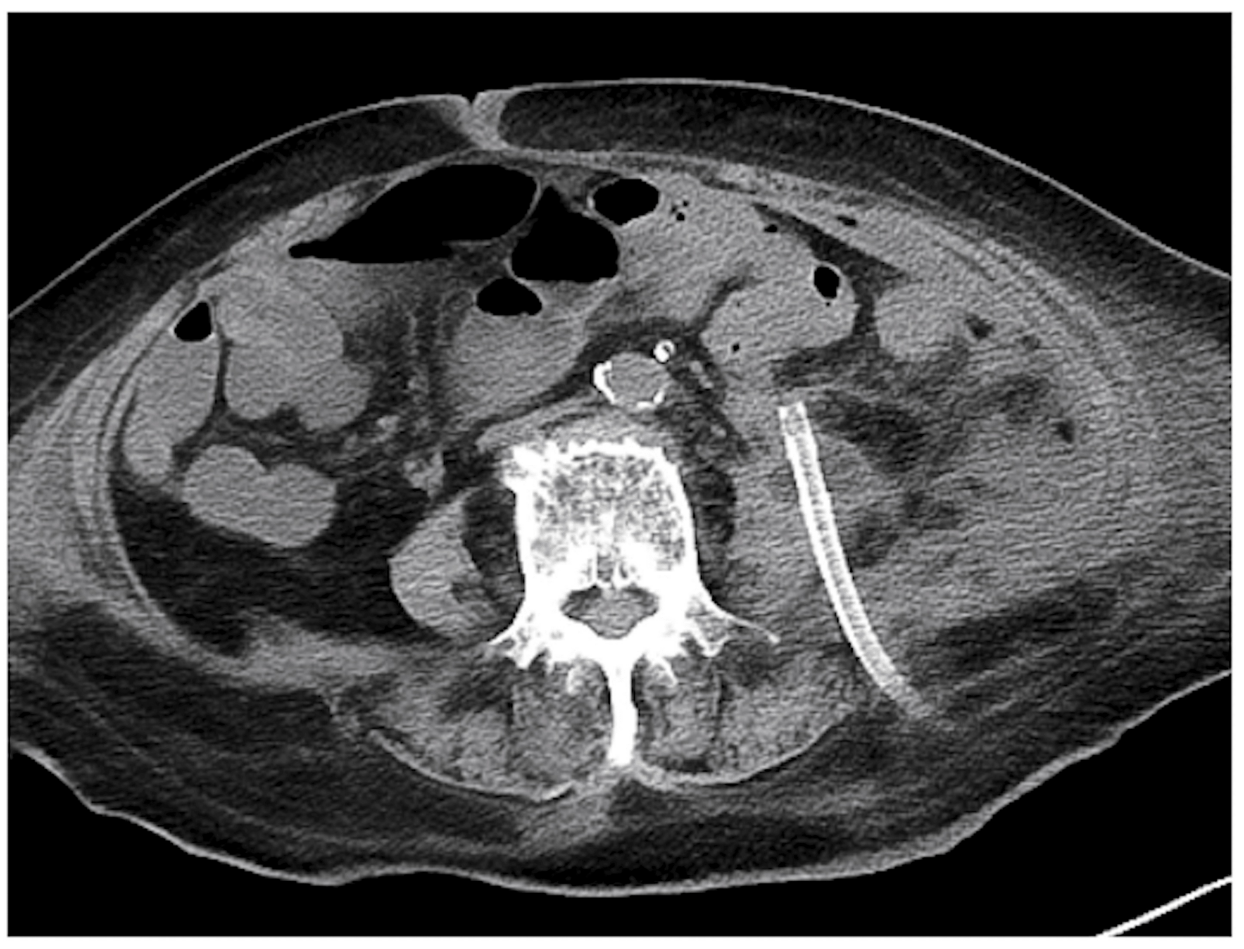
CT scan showing a drainage tube inserted into a retroperitoneal abscess.

On the 61st day of admission, she consulted with the urology department of our hospital. She was transferred to another hospital's urology department for an incisional drainage procedure the next day, as the drainage procedure was difficult to perform in our hospital. After drainage was performed (Figure 4), the patient was again admitted to our hospital and discharged after her condition stabilized.

## Discussion

Bonet first reported spontaneous renal capsular hemorrhage in 1679, Wunderlich first described it clinically in 1856, and Coenen used the term Wunderlich syndrome for this pathology in 1910 [1,5]. The incidence of Wunderlich syndrome ranges from 0.07% to 0.3% and is caused by various etiologies. The most common etiologies include coagulopathy, renal cell carcinoma, renal cysts, renal angiomyolipoma, polycystic kidney disease, and infection [1,3,4]. The incidence of Wunderlich syndrome in infection is 2-10% [3,5-7]. This situation suggests that the cases of Wunderlich syndrome caused by pyelonephritis of pneumococci that we have experienced are rare. However, these studies do not describe the causative agent or treatment of pyelonephritis with Wunderlich syndrome. Therefore, we performed a literature search screening of the Pubmed database using the following keywords: “spontaneous subcapsular renal hematoma” or “Wunderlich syndrome” with “pyelonephritis” in the search engine. Case reports of pyelonephritis causing Wunderlich syndrome are summarized in Table 1 [2,6-14].

**Table 1:** Case reports of pyelonephritis causing Wunderlich syndrome Abx = Antibiotics, CRF = Chronic renal failure, SLE = systemic lupus erythematosus, DM = Diabetes mellitus, HL = Hyperlipidemia, HT = Hypertension, IHD = Ischemic heart disease, LC = Liver cirrhosis, NP = Nephrectomy, PTD = Percutaneous drainage

Auther	Age [Y]	Sex	Comorbidity	Antithrombotic medication	Pathogen	Diagnosis	BP [mmHg]	HR [bpm]	Treatment	Outcome
L. R. Garcia-Chairez [2]	38	F	HT, DM	No	Escherichia coli	CT scan	hypotension, tachycardia		Abx＋NP	Discharge
H. J. Kim [6]	60	F	DM	No	Escherichia coli	CT scan	−	−	Abx＋PTD	Discharge
J. C. H. Chia [7]	70	F	HT, DM, IHD, LC, CRF	Yes	Klebsiella pneumoniae	CT scan	113/89	91	Abx	Discharge
C. F. You [8]	67	F	DM	No	Escherichia coli	CT scan	156/97	102	Abx	Discharge
T. Ozeki [9]	80	F	HT, HL, Cervical spondylosis	No	Escherichia coli	CT scan	103/63	91	Abx＋PTD	Discharge
J. W. Min [10]	57	F	DM, HT, Pyelonephritis	No	Escherichia coli	CT scan	96/52	118	Abx＋PTD	Discharge
J. Mancio [11]	83	F	DM, CRF, Hypothyroidism	No	Klebsiella pneumoniae	CT scan	hypotension, tachycardia		Abx＋NP	Death
L. Dongming [12]	59	F	DM, HT, Renal cyst	No	Burkholderia cepacia	CT scan	85/59	−	Abx＋PTD	Discharge
H. J. Chung [13]	43	F	DM, HT, SLE, Kidney stones	No	Escherichia coli	CT scan	77/54	106	Abx＋PTD	Discharge
T. Bajaj [14]	51	F	DM, Bipolar Disorder	No	Escherichia coli	CT scan	MAP of 55 ~60	120	Abx＋PTD	Discharge
Our hospital	80	F	HT, HL, DM, IHD, Rheumatoid arthritis	Yes	Klebsiella pneumoniae	CT scan	83/42	123	Abx＋PTD	Discharge

Only acute nephritis cases were summarized, excluding xanthogranulomatous pyelonephritis, which is chronic pyelonephritis.

The mean age at which pyelonephritis caused Wunderlich syndrome was 60.8 (43-83) years, and only women were affected. Previous systematic reviews and retrospective studies of Wunderlich syndrome did not show such a sex difference, leading to different results [1,3,4,15]. 

As for comorbidities, all patients had diabetes mellitus, and hypertension was also present in many patients (6/10 cases). Patients with diabetic renal infections are at high risk of developing perirenal hemorrhage, and the results confirm this [16]. Only one case was treated with oral antithrombotic medication, and most of the patients were bleeding even without oral antithrombotic medication. 

CT is most helpful for diagnosis. It allows examination of the kidneys and adjacent organs and often provides a presumption of etiology [7]. The CT scan was used in all cases we examined.

The treatment of Wunderlich syndrome depends on the clinical situation, with hemodynamically stable patients receiving conservative treatment and hemodynamically unstable patients requiring surgical intervention: embolization or nephrectomy [2]. 

Conservative treatment in pyelonephritis causing Wunderlich syndrome primarily means treatment with antimicrobial agents only. Table 1 shows that the causative organisms of pyelonephritis causing Wunderlich syndrome were *Escherichia coli* in seven cases (70%), *Klebsiella pneumoniae *in two cases (20%), and *Burkholderia cepacian* in one case (10%). Antimicrobial agents that mainly cover Gram-negative rods should be administered. It should be noted that there were also cases of extended-spectrum beta-lactamase-producing *E. coli *[2].

However, Table 1 shows that Wunderlich syndrome due to acute pyelonephritis was treated with antibacterial agents and percutaneous drainage or nephrectomy in 80% of cases (8/10) and with antibiotics alone in only two cases. Of the eight patients who underwent percutaneous drainage or nephrectomy, six had shock vitals at presentation, indicating that Wunderlich syndrome due to acute pyelonephritis is prone to hemodynamic instability. In some cases, the infection progressed rapidly in the presence of pre-existing diabetes, leading to emphysematous pyelonephritis and necrotizing fasciitis [10,12]. In our case, the patient had diabetes mellitus and rheumatoid arthritis, which led to the formation of an abscess in the erector spine muscle, requiring drainage.

## Conclusions

We report a case of Wunderlich syndrome caused by pyelonephritis due to *Klebsiella pneumonia*. Wunderlich syndrome caused by pyelonephritis is often associated with pre-existing diabetes mellitus and shock vitals, and it is important to keep in mind that surgical treatment (percutaneous drainage or nephrectomy) may be performed in addition to antimicrobial agents.
